# Selenium intake modifies the association of dietary fat with hypertension among Chinese adults: a cohort study

**DOI:** 10.3389/fnut.2026.1733016

**Published:** 2026-03-03

**Authors:** Jia-min Yan, Min-zhe Zhang, Si-jing Liu, Hui Li, Qiang Li, Hong-jie Yu, Qi-qiang He, Bang-xian Ding, Ye Zeng

**Affiliations:** 1 Department of Laboratory Medicine, Wuhan Children’s Hospital (Wuhan Maternal and Child Healthcare Hospital), Tongji Medical College, Huazhong University of Science & Technology, Wuhan, China; 2 School of Public Health, Wuhan University, Wuhan, China; 3 Children’s Medical Ward I, Wuhan Children’s Hospital (Wuhan Maternal and Child Healthcare Hospital), Tongji Medical College, Huazhong University of Science & Technology, Wuhan, China; 4 Medical Department, Taixing People’s Hospital, Jiangsu, China; 5 School of Health and Nursing, Wuchang University of Technology, Wuhan, China; 6 School of Public Health, Wuhan University, Wuhan, China; 7 Hubei Biomass-Resource Chemistry and Environmental Biotechnology Key Laboratory, Wuhan University, Wuhan, China

**Keywords:** cohort, fat intake, hypertension, modifying effects, selenium intake

## Abstract

**Background:**

The role of selenium intake on the association of fat intake with hypertension remains unclear. This study aimed to investigate the relationship among selenium intake, dietary fat and hypertension risk among Chinese adults.

**Methods:**

Data from the China Health and Nutrition Survey were used. Individuals who were free of hypertension at baseline (2004) and participated at least once in the subsequent surveys (2006, 2009, and 2011) were included in this study. Generalized estimating equation models were used to explore the relationship between fat intake and systolic and diastolic blood pressure, and hypertension risk, as well as the modifying effect of selenium intake.

**Results:**

Among a total 5,643 participants, 1,722 adults developed hypertension during the follow-up period. After adjusting for covariates, participants in the highest fat intake group had significantly increased risk of hypertension compared with the lowest group (OR: 1.22, 95% CI: 1.01–1.47). The risk of hypertension tended to increase with increasing fat intake in the low selenium intake group, but not in the high selenium intake group. Systolic blood pressure increased by 0.69 mmHg (95% CI: 0.17–1.22) with increasing 50 g/day fat intake in the low selenium intake group (T1), whereas this association was not significant in the selenium intake T2 and T3 groups.

**Conclusion:**

Selenium intake may modify the relationship between fat intake and hypertension, with high selenium intake attenuating the negative effects of fat intake on hypertension risk.

## Introduction

Hypertension is a crucial risk factor for chronic kidney disease, cardiovascular disease and premature mortality, emerging as a prominent global public health issue (1). The rate of hypertension continues to rise quickly in low and middle-income countries (2). A latest national survey reported that hypertension affects 23.2% of Chinese population aged 18 years and above, equating to approximately 240 million individuals (3). Despite the development of high-quality management and treatment strategies, these measures usually cannot effectively solve the problem (4). Therefore, it is crucial to investigate the influencing factors for the primary prevention of hypertension.

Emerging research has increasingly focused on the relationship between dietary intake and hypertension risk (5, 6). Among several dietary factors, selenium (Se), one indispensable trace element for human health, has antioxidant capabilities and is capable of safeguarding cells from the damage by free radicals and oxidative stress conditions (7). In contrast, high dietary fat intake, especially saturated fat, may induce an inflammatory response in tissues, which has been linked to vascular lesions, leading to high blood pressure (8, 9). Although some studies showed an increased risk for hypertension in individuals with high dietary total fat and low Se intake (10, 11), others found a negative association of total fat or no relationship of Se with hypertension (12, 13). Furthermore, no longitudinal research has examined the synthetic effect of dietary fat and Se intake on the risk of hypertension. Therefore, we investigated the relationship of dietary fat with hypertension and the possible modifying effect of Se intake among a cohort of Chinese adults.

## Methods

Data was used from the China Health and Nutrition Survey (CHNS), a prospective cohort study to collect comprehensive information on the health and nutritional status of Chinese population over time (14). This study was approved by the Institutional Review Boards at the University of North Carolina at Chapel Hill and the National Institute for Nutrition and Health and the Chinese Center for Disease Control and Prevention. All participants provided written informed consent (15). This study adhered to the STROBE-nut guidelines.

The current research included participants aged 18 years and above who did not have hypertension in the baseline survey (2004) and participated at least once in the subsequent studies (2006, 2009, and 2011). Participants who did not provide information on blood pressure and significant covariate data were excluded. Finally, 5,643 participants were included in the current study.

During a 24-h dietary review survey, trained investigators recorded the specific types and quantities of all foods consumed by participants over three-days (including 2 weekdays and 1 weekend day). The total fat, Se, sodium (Na) and potassium (K) intakes were calculated according to the Chinese Food Composition Table (16).

In preparation for blood pressure evaluation, participants were asked to sit for a duration of 10 min to ensure a stable reading. Subsequently, blood pressure measurements were taken by medical staff utilizing a conventional mercury sphygmomanometer. The measurement procedure involved positioning the cuff on the participant’s right arm, ensuring that the bottom edge of the cuff was situated 25 millimeters proximal to the elbow. To ensure accuracy, blood pressure was measured three times, and the mean value was documented for further analysis. Participants were considered to develop hypertension during the follow-up if they met any one of four established criteria: (1) Receiving a new medical diagnosis of hypertension, (2) initiation of anti-hypertensive medication treatment, (3) systolic blood pressure (SBP) ≥ 140 mmHg, or (4) Diastolic blood pressure (DBP) ≥ 90 mmHg (17).

Information on demographics and lifestyle, including age, gender, education, residence, physical activity, alcohol consumption, smoking, and energy intake, were collected through self-administered questionnaires. Body Mass Index (BMI) was calculated by dividing an individual’s body weight by the square of their height (kg/m^2^). The classification of residency varied from city to suburban, town or county capital, and rural village. Education was divided as primary school and below, middle school, high school and above. Smoking status was categorized into three groups: current smokers, who encompassed both actively smoking and recent quitters with a cessation period of less than 12 months; former smokers, who had discontinued smoking for over 12 months; and never smokers. Individuals were considered current alcohol drinkers if they reported consuming alcohol more frequently than three times weekly. Physical activity was assessed based on self-reported daily activities and their corresponding Metabolic Equivalent of Task (MET) values, which encompassed activities related to transportation, periods of sedentariness, leisure-time physical exertion, and occupational tasks. The total energy intake for each participant was ascertained by aggregating the caloric values of all foods consumed.

Differences between participants with hypertension and those without were assessed by t test or Chi-square test. The participants were divided into three subgroups (lowest T1 to highest T3) according to the tertile of Se intake and five groups (lowest Q1 to highest Q5) according to the quintile of fat intake in all waves. The interaction effect was investigated by incorporating an interaction variable, which represented the combined effect of Se and fat intake, into the regression model. Given a significant interaction was observed, we conducted a stratified analysis and presented the results by Se intake and fat intake.

Logistic and linear generalized estimation equation (GEE) models were employed to assess the relationship of total fat intake with hypertension, and the levels of SBP and DBP of participants and subgroups by Se intake. Two models were utilized to investigate the effects of demographic and lifestyle factors. Model 1 was adjusted for age, gender, smoking and alcohol consumption. Model 2 was additionally adjusted for residence, education, physical activity, BMI, energy intake, sodium and potassium intake. All statistical analyses were carried out by R software (version 4.3.1, http://www.R-project.org/).

## Results

Among a total of 5,643 participants, 1,722 were diagnosed with hypertension during the follow-up period. The mean age (SD) was 45.5 (13.5) years old, and 2,540 (45%) were males. Hypertensive participants were older, had significantly higher energy intake and lower Se intake, and were more likely to be male, overweight/obesity, rural residence, low education, alcohol drinker, smokers, and had low physical activity than their counterparts (Table 1).

**Table 1:** Baseline characteristics of participants.

Characteristics	Total (*N* = 5,643)	Noncases (*N* = 3,921)	Cases (*N* = 1722)	*p*-value
Age, years	45.5 ± 13.5	42.9 ± 13.0	55.7 ± 12.6	<0.001
Gender, *n* (%)				<0.001
Male	2,540 (45.0)	1,697 (43.3)	843 (49.0)	
Female	3,103 (55.0)	2,224 (56.7)	879 (51.0)	
BMI, *n* (%)				<0.001
<18.5 kg/m^2^	355 (6.3)	285 (7.3)	70 (4.1)	
18.5–24 kg/m^2^	3,544 (62.8)	2,607 (66.5)	937 (54.4)	
24–28 kg/m^2^	1,440 (25.5)	883 (22.5)	557 (32.3)	
>28.0 kg/m^2^	304 (5.4)	146 (3.7)	158 (9.2)	
Residence, *n* (%)				0.003
City	711 (12.6)	515 (13.1)	196 (11.4)	
Suburban	1,116 (19.8)	812 (20.7)	304 (17.7)	
Town or county capital city	808 (14.3)	563 (14.4)	245 (14.2)	
Rural village	3,008 (53.3)	2031 (51.8)	977 (56.7)	
Education, *n* (%)				<0.001
Primary and below	2,394 (42.4)	1,479 (37.7)	915 (53.1)	
Junior high	2,706 (48.0)	2026 (51.7)	680 (39.5)	
Senior high and above	543 (9.6)	416 (10.6)	127 (7.4)	
Alcohol consumption, *n* (%)				<0.001
No	4,850 (85.9)	3,440 (87.7)	1,410 (81.9)	
Yes	793 (14.1)	481 (12.3)	312 (18.1)	
Smoking, *n* (%)				<0.001
Never	3,874 (68.7)	2,749 (70.1)	1,125 (65.3)	
Former	91 (1.6)	51 (1.3)	40 (2.3)	
Current	1,678 (29.7)	1,121 (28.6)	557 (32.3)	
Energy intake, kcal/day	2233.5 ± 661.8	2221.5 ± 651.3	2261.1 ± 684.6	0.042
Physical activity, MET-hours/week	126.8 ± 126.0	130.8 ± 126.8	117.8 ± 123.9	<0.001
Fat intake, g/day	69.6 ± 37.8	69.1 ± 37.4	70.5 ± 38.6	0.199
Selenium intake, μg/day	47.1 ± 34.9	47.9 ± 37.5	45.4 ± 28.0	0.006
Sodium intake, g/day	5.8 ± 5.4	5.8 ± 5.3	6.0 ± 6.2	0.151
Potassium intake, g/day	1.8 ± 0.9	1.8 ± 0.8	1.9 ± 1.2	0.002

BMI: Body Mass Index.

Table 2 displays the relationship between dietary fat and hypertension by Se intake. Results show that fat intake was correlated with a significantly increased risk of hypertension in Q3, Q4, and Q5 groups (*p* < 0.05). Stratified analysis revealed that in the Se T1 group, the OR (95% CI) of hypertension was 1.49 (1.15–1.95) in Q3, 1.31 (1.01–1.76) in Q4, and 1.52 (1.11–2.10) in Q5 dietary fat group, respectively. However, the relationship was attenuated with the increasing Se intake. In Se T2 group, the statistically significant association was only found in the Q5 dietary fat group (OR: 1.40, 95% CI: 1.00–1.95), while no significant relationship was found in the Se T3 group.

**Table 2:** The association between fat intake and hypertension, stratified by Se intake.

Variables	Quintile of fat intake				
	Q1	Q2	Q3	Q4	Q5
All respondents					
Range (g/day)	<37.9	37.9–55.1	55.1–73.1	73.1–97.0	>97.0
*N*, total	1,127	1,130	1,129	1,129	1,128
Model 1	Ref	1.11 (0.94–1.31)	1.28 (1.08–1.50)*	1.22 (1.03–1.44)*	1.27 (1.07–1.49)*
Model 2	Ref	1.11 (0.94–1.31)	1.27 (1.08–1.51)*	1.21 (1.01–1.44)*	1.22 (1.01–1.47)*
Se intake T1 (<32.2 μg/day)					
*N*, total	670	479	367	218	147
Model 1	Ref	1.17 (0.90–1.52)	1.60 (1.23–2.08)*	1.45 (1.09–1.92)*	1.89 (1.42–2.51)*
Model 2	Ref	1.13 (0.87–1.46)	1.49 (1.15–1.95)*	1.31 (1.01–1.76)*	1.52 (1.11–2.10)*
Se intake T2 (32.2–50.2 μg/day)					
*N*, total	275	419	418	436	333
Model 1	Ref	1.00 (0.75–1.35)	1.31 (0.99–1.74)	1.09 (0.82–1.46)	1.34 (1.01–1.78)*
Model 2	Ref	1.04 (0.76–1.42)	1.26 (0.93–1.70)	1.22 (0.90–1.66)	1.40 (1.00–1.95)*
Se intake T3 (>50.2 μg/day)					
*N*, total	183	231	344	475	648
Model 1	Ref	1.03 (0.78–1.37)	0.94 (0.70–1.25)	1.01 (0.77–1.34)	0.81 (0.60–1.08)
Model 2	Ref	1.13 (0.81–1.57)	0.99 (0.71–1.37)	0.99 (0.71–1.38)	0.84 (0.60–1.17)

*p < 0.05.

Model 1: adjusted for age, gender, smoking, alcohol consumption.

Model 2: adjusted for age, gender, BMI, energy intake, residence, education, smoking, alcohol consumption, physical activity, sodium intake, potassium intake.

The association between the fat intake per 50 g/day increment and hypertension, SBP, and DBP by Se intake is shown in Table 3. In the total participants, a significantly increased risk of hypertension (OR: 1.07, 95% CI: 1.00–1.15) was observed with each 50 g/day fat increment. In the stratified analysis by Se intake, each 50 g/day fat intake was associated with an increased risk of hypertension (OR: 1.19, 95% CI: 1.05–1.35), increased SBP (*β*: 0.69, 95% CI: 0.17–1.22) in the low Se intake group (T1). Nevertheless, there was no significant relationship between fat intake and hypertension and SBP among participants with high Se intake (T3).

**Table 3:** The association of fat intake (per 50 g/day increment) with hypertension, SBP, and DBP, stratified by Se intake.

Variable	All respondents		Tertile of Se intake					
			T1		T2		T3	
	OR/*β*	*p*	OR/*β*	*p*	OR/*β*	*p*	OR/*β*	*p*
Hypertension^a^								
Model 1	1.08 (1.02–1.15)	0.006	1.28 (1.13–1.45)	<0.001	1.11 (1.00–1.23)	0.047	0.90 (0.78–1.04)	0.170
Model 2	1.07 (1.00–1.15)	0.047	1.19 (1.05–1.35)	0.008	1.16 (1.02–1.33)	0.022	0.88 (0.77–1.02)	0.089
SBP^b^								
Model 1	0.22 (−0.03–0.48)	0.090	0.85 (0.34–1.36)	0.001	0.09 (−0.39–0.57)	0.714	−0.29 (−0.78–0.20)	0.249
Model 2	0.06 (−0.22–0.34)	0.667	0.69 (0.17–1.22)	0.010	0.10 (−0.44–0.64)	0.716	−0.51 (−0.90–0.12)	0.151
DBP^b^								
Model 1	0.08 (−0.16–0.32)	0.516	0.32 (0.02–0.62)	0.037	0.12 (−0.22–0.46)	0.485	−0.09 (−0.44–0.27)	0.628
Model 2	0.01 (−0.17–0.19)	0.193	0.20 (−0.10–0.49)	0.190	0.13 (−0.24–0.50)	0.497	−0.27 (−0.55–0.10)	0.104

SBP, systolic blood pressure; DBP, diastolic blood pressure.

Model 1: adjusted for age, gender, smoking, alcohol consumption.

Model 2: adjusted for age, gender, BMI, energy intake, residence, education, smoking, alcohol consumption, physical activity, sodium intake, potassium intake.

a OR for hypertension.

b *β* for SBP and DBP.

## Discussion

In this prospective study of 5,643 Chinese adults, a significant positive relationship was found between dietary total fat intake and hypertension. In addition, high Se intake could attenuate the effect of fat intake on hypertension, with a significant association of fat intake and hypertension risk only observed in participants with low Se intake.

Epidemiological evidence has shown that over-consumption of dietary fatty acids was significantly associated with multiple health outcomes (18). However, findings on the relationship between dietary total fat and hypertension are inconclusive. Currenti et al. (19) reported that high intakes of total fat were associated with a significantly decreased risk of hypertension in a cohort of 1936 Italian adults, while a meta-analysis of 43 cohort studies showed no significant association of total fat with cardiovascular diseases (including hypertension) (12). Nevertheless, findings of our study are consistent with the study by Appel et al. (10), who found that a diet with reduced total fat can substantially lower SBP and DBP. Dietary fats include several subtypes-saturated fatty acid (SFA), monounsaturated fatty acid (MUFA), polyunsaturated fatty acid (PUFA), and trans-fatty acid (TFA), showing different linkage with blood pressure and hypertension risk (20). It has been reported that SFAs were the major compounds among dietary fats in Chinese adults, and higher intake of SFA may induce inflammatory and oxidative stress that contribute to the development of hypertension (21). This might explain the increased risk of hypertension among our participants with high total fat intake. However, more studies are warranted to examine the effects of total and subtypes of dietary fat on the risk of hypertension.

Se is a necessary trace element for human health through a wide range of biological functions, including immune and antioxidant defense, organ protection, and cardiovascular regulation, etc. Se has been closely associated with cardiovascular diseases, cancers, mortality (22). However, some studies show a negative relationship of dietary Se intake with hypertension (11). A meta-analysis of randomized control trials (RCTs) found that Se supplementation significantly increased SBP (23), while another meta-analysis of 16 prospective studies and 16 RCTs suggested a nonlinear relationship between Se and CVD risk, and the significant inverse association was observed within a narrow Se range (24). Se compounds can be categorized into inorganic (such as selenates and selenites) and organic forms (such as selenoproteins and selenoamino acids). As plant-derived organic Se compounds show significantly higher bioavailability and lower toxicity than inorganic Se compounds, this may partly explain the different results among previous studies. In the present study, we found that dietary Se intake above 50.2 μg/day significantly attenuated the risk of hypertension among participants with high dietary fat intake. Our results lend support to the dietary recommendation for Se (55 μg /day for both men and women) (25). Therefore, our findings suggest that adequate dietary Se intake and reducing total fat are recommended to lower hypertension risk synergistically in Chinese adults.

The metabolic fate and bioavailability of Se depend on its chemical form. Once absorbed, both organic and inorganic Se compounds are ultimately converted into low-molecular-weight Se species, which are precursors for selenoprotein synthesis. Thus, plant-derived Se compounds are ideal dietary sources for the purpose of anti-aging, anticancer, and other health-promotion. Besides, organic Se and inorganic forms undergo distinct biotransformations but converge at the production of hydrogen selenide, a key intermediate. To prevent toxicity, excess selenides are detoxified through methylation to form metabolites like trimethylselenonium for urinary excretion, or converted into selenosulfides (26, 27). These intricate metabolic and detoxification processes ensure that Se levels remain within a physiological range to exert antioxidative effects without inducing toxicity. This regulatory mechanism ensures the maintenance of Se within a narrow and safe range. Recent meta-analyses have further emphasized that serum Se levels were intricately linked to lipid profiles, suggesting that Se status may modulate how the body processes dietary fats (28).

To the best of our knowledge, this is the first study to explore the modifying effect of Se intake on the association between dietary fat and hypertension. Several biochemical mechanisms might explain the interaction effect of Se and fat intake on hypertension risk. Dietary fat may contribute to the onset and progression of hypertension via increased inflammatory response and oxidative stress (21). In contrast, Se is an essential trace element that is incorporated into selenoproteins, including glutathione peroxidases (GPx), thioredoxin reductases (TrxR), and selenoprotein P. These enzymes play a fundamental role in neutralizing reactive oxygen species (ROS) and regulating redox homeostasis, thereby inhibiting the oxidative damage to lipids and preventing atherosclerotic plaque formation caused by high fat intake (29, 30). Furthermore, Se plays a pivotal antioxidative role in immune cell signaling to reduce inflammation and affect the immune system (31). Another mechanism could be attributed to the regulation effects of Se on the intestinal microbiota. High fat diet has been associated with lower richness and diversity of the intestinal microbiota in human body, which have shown an important role in the pathogenesis of hypertension (32). Se also shows specific antibacterial activity to affect the composition of the intestinal microbiota and gastrointestinal tract colonization, thus attenuating the negative effects of high dietary fat on blood pressure (33).

There are several limitations in this study. Firstly, dietary assessment was evaluated using a 24-h recall method, thus recall bias may exist. Secondly, although several potential confounders have been controlled, residual confounding effects by unmeasured factors could not be excluded. Thirdly, the effects of different types of dietary fat and the combined effects with Se on hypertension risk remain unknown. Fourthly, biomarkers of oxidative stress and inflammation were not available in the CHNS, limiting our ability to directly verify the proposed pathways. Fifthly, detailed information on fatty acid subtypes (e.g., SFA, MUFA, and PUFA) was available for only a subset of food items, which precluded a robust assessment of fatty acid quality. Lastly, our study cohort consists entirely of Chinese adults, which limits the generalizability of the findings to populations with different dietary patterns. Chinese dietary patterns, characterized by specific cooking oils and fat sources, differ substantially from the Mediterranean or Western diets (34). Furthermore, Se intake varies significantly across countries. For example, the United States and Japan have higher Se intake levels, while Chinese population reported lower intake (35). Thus, future multi-ethnic studies are necessary to validate these results across diverse geographical and nutritional contexts.

In conclusion, the present study suggests that high fat intake diet may increase the risk of hypertension, whereas Se intake could attenuate the dietary fat-hypertension relationship in Chinese adults. Our results support the dietary recommendations on optimal amount of food fat and Se intake for hypertension prevention.

## Data Availability

Publicly available datasets were analyzed in this study. This data can be found at: https://chns.cpc.unc.edu/. Requests to access the data should be directed to chns@unc.edu.
